# Tumor Necrosis Factor α and Regulatory T Cells in Oncoimmunology

**DOI:** 10.3389/fimmu.2018.00444

**Published:** 2018-03-12

**Authors:** Benoît L. Salomon, Mathieu Leclerc, Jimena Tosello, Emilie Ronin, Eliane Piaggio, José L. Cohen

**Affiliations:** ^1^Sorbonne Université, INSERM, CNRS, Centre d’Immunologie et des Maladies Infectieuses (CIMI-Paris), Paris, France; ^2^Université Paris-Est and INSERM U955, Créteil, France; ^3^Service d’Hématologie Clinique et de Thérapie Cellulaire, Assistance Publique Hôpitaux de Paris (APHP), Hôpital H. Mondor, Créteil, France; ^4^Center of Cancer Immunotherapy and Centre d’Investigation Clinique Biothérapie 1428, Institut Curie, PSL Research University, INSERM U932, Paris, France; ^5^Centre d’Investigation Clinique Biothérapie, Assistance Publique Hôpitaux de Paris (APHP), Hôpital H. Mondor, Créteil, France

**Keywords:** tumor necrosis factor α, TNFR2, regulatory T cells, cancer, graft-versus-host disease, immunotherapy

## Abstract

Tumor necrosis factor α (TNF) is a potent pro-inflammatory cytokine that has deleterious effect in some autoimmune diseases, which led to the use of anti-TNF drugs in some of these diseases. However, some rare patients treated with these drugs paradoxically develop an aggravation of their disease or new onset autoimmunity, revealing an immunosuppressive facet of TNF. A possible mechanism of this observation is the direct and positive effect of TNF on regulatory T cells (Tregs) through its binding to the TNF receptor type 2 (TNFR2). Indeed, TNF is able to increase expansion, stability, and possibly function of Tregs *via* TNFR2. In this review, we discuss the role of TNF in graft-versus-host disease as an example of the ambivalence of this cytokine in the pathophysiology of an immunopathology, highlighting the therapeutic potential of triggering TNFR2 to boost Treg expansion. We also describe new targets in immunotherapy of cancer, emphasizing on the putative suppressive effect of TNF in antitumor immunity and of the interest of blocking TNFR2 to regulate the Treg compartment.

## TNFR2 on Regulatory T Cell (Treg): State of the Art

### Immunosuppressive Feature of Tumor Necrosis Factor α (TNF)

Tumor necrosis factor α is a pleiotropic cytokine produced by various cell types and involved in a wide range of pathological processes [for review, see Ref (1, 2)]. It has been initially considered as a pro-inflammatory molecule. However, preclinical and clinical data have shown that it also mediates a paradoxical anti-inflammatory and immunomodulatory effect. Indeed, in murine models of type 1 diabetes or lupus nephritis, TNF may have a protective effect (3–7). Moreover, new onset or exacerbation of chronic inflammatory and autoimmune diseases has been observed in patients treated with anti-TNF therapies (8–14). We will describe below in detail the case of graft-versus-host disease (GVHD) as an example of the ambivalent action of TNF in an immunopathology.

### Different Possible Mechanisms for the Suppressive Action of TNF

**Figure 1:**
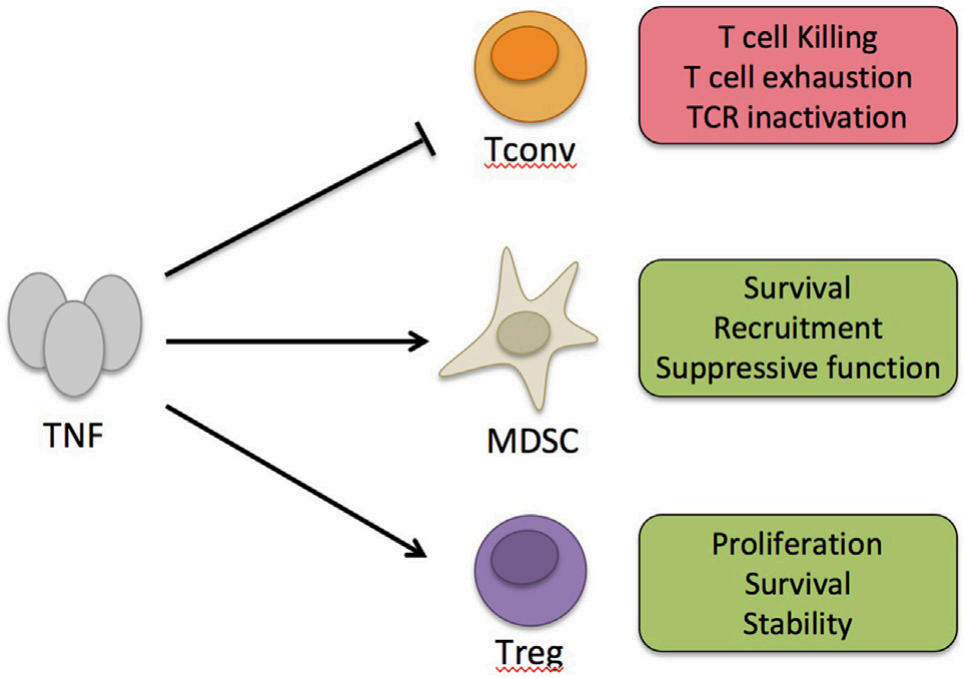
Immunosuppressive action of tumor necrosis factor α (TNF). TNF can exert its immunosuppressive activity by intrinsic negative effect on conventional T cells (Tconvs) activation or by boosting suppressive cells, such as myeloid-derived suppressor cells (MDSC) or regulatory T cells (Tregs). On Tconvs, long-term effect of TNF may promote killing, exhaustion, or TCR inactivation. On MDSC, TNF may boost their activity by promoting their survival, local recruitment, or suppressive function. On Tregs, TNF may promote their proliferation, survival, and stability.

Tumor necrosis factor α binds to two receptors, namely, TNF receptor type 1 (TNFR1) and TNFR2 (Figure 1). Unlike TNFR1 that has a ubiquitous expression, TNFR2 is expressed by some immune cells, preferentially by a fraction of Tregs, some endothelial cells, and cells of the nervous tissue (2, 15). Several mechanisms have been proposed to explain the suppressive action of TNF. It was shown that chronic stimulation with TNF may inactivate TCR signaling (16) or induce T cell exhaustion (17). Alternatively, the cytokine may kill CD8+ T cells, a phenomenon emphasized for autoreactive cells (18). Besides these cell-intrinsic mechanisms, TNF may exert its suppressive activity by stimulating cells that have immunosuppressive action, such as myeloid-derived suppressor cells (MDSCs) (19, 20). Finally, the pioneer works of Chen and Oppenheim suggested that this immunosuppressive effect of TNF could be related to a direct activation of Tregs (15, 21). This latter mechanism, which is the most studied one and supported by data obtained by different groups, is detailed below. Generally, the suppressive action of TNF is considered to be mediated by its interaction with TNFR2 since TNFR2 signaling appears to be protective in various immunopathologies and several of the mechanisms described earlier are TNFR2 dependent (22). However, whereas TNF/TNFR1 interaction has been mostly described to be pro-inflammatory, TNFR1 triggering may also inhibit IL-12/IL-23 p40 expression by macrophages (23). This mechanism may explain the paradoxical expansion of Th1/Th17 cells following anti-TNF treatment in patients with autoimmune diseases who do not respond to this therapy (24, 25).

### TNFR2 Expression by Tregs

TNFR2 expression is upregulated in activated Tregs and can be detected in activated conventional T cells (Tconvs), although at lower levels than in activated Tregs. Some other members of the TNFR family, such as GITR, OX40, or 4-1BB, are also preferentially expressed by Tregs, and their expression is also upregulated upon activation (26). Remarkably, in transcriptomic analyses, comparing Tregs and Tconvs of lymphoid tissues, TNFR2, OX40, and GITR belong to the Treg signature and their expression correlates with low DNA methylation in Tregs suggesting that their transcription is at least partly regulated at the epigenetic level (27, 28). These three molecules are expressed early in the Treg lineage, since the thymic Treg progenitor stage, and their expression is essential for Treg development (29). In mice lymphoid tissues or in human blood, TNFR2 is expressed by the fraction of activated Tregs expressing high levels of other activation markers such as CTLA-4 (30). TNFR2 expression remarkably identifies a subset of Tregs with the highest suppressive capacity (21, 30, 31).

### Stimulating Effect of TNF on Tregs *via* TNFR2

The direct effect of TNF on TNFR2-expressing Tregs has been studied by Chen and Oppenheim *in vitro* and has been reviewed elsewhere (32). Briefly, TNF increases proliferation, survival, stability, expression of CD25, Foxp3, and activation markers, as well as suppressive function of mouse Tregs (15, 26, 30, 31). Many of these effects of TNF, notably on proliferation, could be reproduced with human Tregs (32–35). However, some studies claim that TNF inhibits the suppressive activity of human Tregs (36–39). The interpretation of some of these studies was complicated by the fact that TNF can render Tconvs more refractory to the Treg-mediated suppression. After extensive and careful exploration of this question, we could conclude that TNF does not inhibit the suppressive activity of human Tregs (35).

### Role of TNFR2 on Treg Biology *In Vivo*

The *in vivo* role of TNFR2 on Treg biology has been more difficult to evaluate because of the absence of a conditional knockout of TNFR2 in Tregs. However, there is strong evidence that TNF can boost Treg expansion in different inflammatory contexts (40). We showed that TNF, probably produced by Tconvs, stimulated Treg proliferation during type 1 diabetes (41). Others observed a similar phenomenon during septic shock, infectious disease, or immune response (15, 42, 43). Also, TNFR2-deficient Tregs lost their capacity to control colitis, which was associated with reduced survival and stability compared with wild-type control Tregs (31, 44). The critical role of TNFR2 expressed by Tregs has been also studied in the context of GVHD and cancer and will be specifically discussed below. Overall, among all the effects of TNF on Treg biology, its capacity to increase proliferation is the most convincing since it has been reported in many *in vitro* and *in vivo* studies performed by different groups using mouse and human Tregs. The evidence that this cytokine also increases Treg survival and stability is quite convincing and its effect on Treg function requires further investigation.

## Hope and Disappointment in Targeting TNF in GVHD

### TNF and TNFR1 As Predictive Biomarkers in GVHD

**Figure 2:**
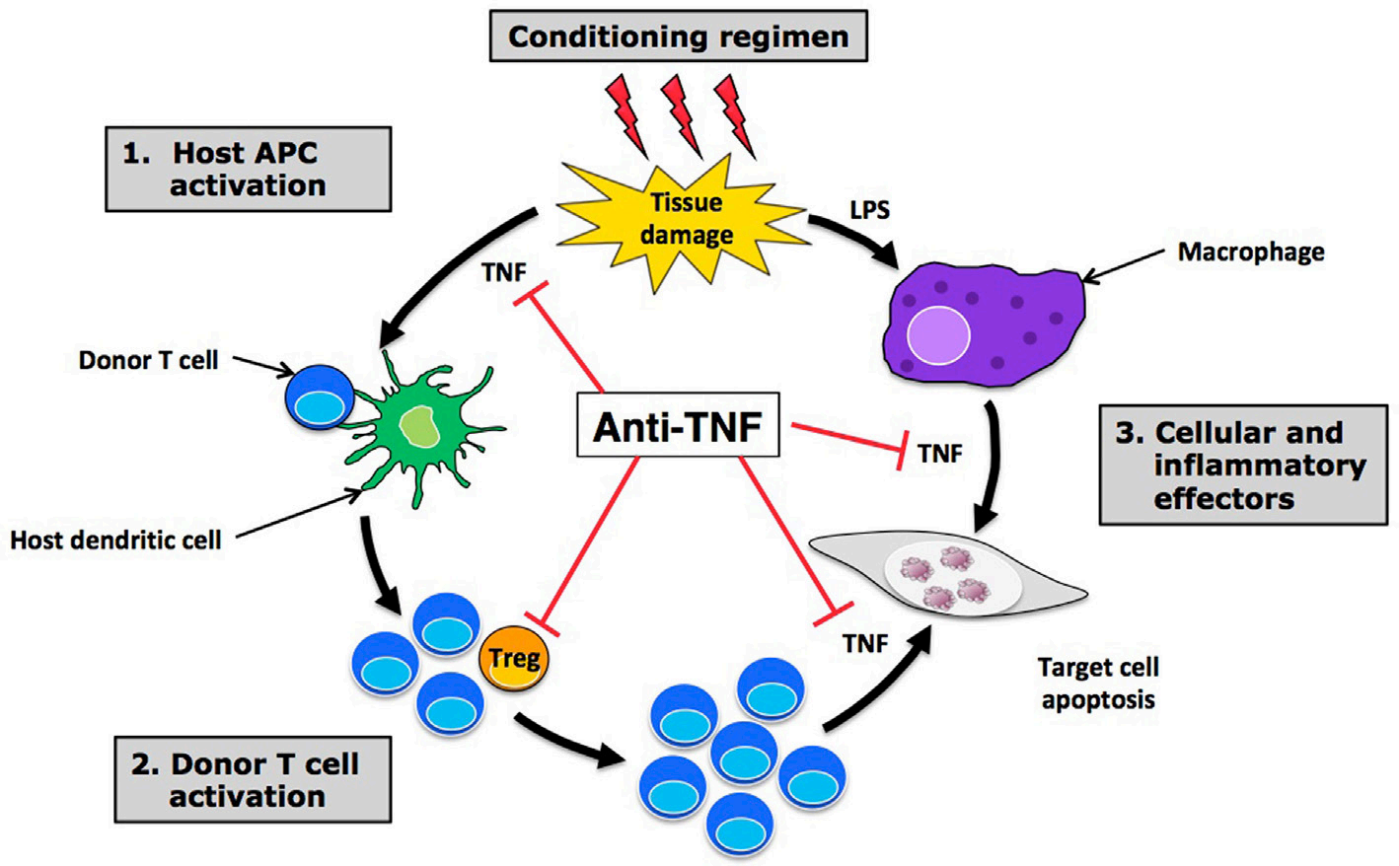
Hope and disappointment in targeting tumor necrosis factor α (TNF) in graft-versus-host disease (GVHD). Anti-TNF treatments are able to block the effect of TNF at different steps of acute GVHD pathophysiology, including initial host APC activation (1), effector T cell recruitment and activation in target tissues (2), and direct cell necrosis (3). By inhibiting TNF ligation to TNFR2 expressed by regulatory T cells (Tregs), anti-TNF treatments could also have a deleterious effect on these suppressive cells, leading to an increased expansion and activation of alloreactive donor T cells that may be responsible for the disappointing results observed with anti-TNF treatments in this setting. Abbreviations: APC, antigen-presenting cell; LPS, lipopolysaccharide.

Tumor necrosis factor α plays a key role in acute GVHD (aGVHD), a systemic and highly inflammatory complication that occurs after allogeneic hematopoietic stem cell transplantation (allo-SCT) (45). TNF indeed plays a major role at different steps of this pathological process in which donor T cells recognize as foreign host healthy tissues and eventually cause their destruction (Figure 2). In this line, clinical studies have clearly demonstrated a positive correlation between soluble TNFR1 levels measured 7 days after transplant and the time to onset and severity of aGVHD (46, 47). The increase in TNFR1 levels between baseline and day 7 was not only an independent predictor of aGVHD but also of transplant-related mortality and overall survival. Also, a rise in TNF, as measured by protein levels in peripheral blood, RNA transcription levels, or flow cytometry, precedes the onset of aGVHD, before peaking at the time of its development (48–50). Overall, the results of these clinical studies have led to the integration of TNFR1 as part of a biomarker panel that can discriminate patients with and without aGVHD, and predict survival (51).

### Anti-TNF Clinical Trials in GVHD

The key role of TNF in aGVHD pathophysiology logically led researchers and physicians to try to block this cytokine to decrease inflammation and consequently to prevent or treat aGVHD. Along this line, most of the clinical trials focused on two molecules: infliximab—a monoclonal antibody (mAb) that binds TNF—and etanercept—a soluble TNFR that competes with cellular receptors for TNF binding. The great hope risen by TNF targeting in aGVHD during the first decade of this century has unfortunately faded rapidly due to somewhat disappointing results of clinical studies. Indeed, clinical trials failed to prove any benefit in adding infliximab to standard treatment, both for aGVHD prophylaxis and treatment (52, 53). Only small retrospective studies have shown promising response rates for the treatment of steroid-refractory aGVHD, mostly in case of intestinal tract involvement (54–57). However, the benefit of infliximab in steroid-refractory aGVHD does not seem to be superior to the one observed with other drugs available (58), even though prospective randomized trials are lacking. Moreover, other studies have shown that responses after infliximab therapy are poorly sustained and have raised concern over a heightened risk of severe infections (59, 60).

Regarding etanercept, a single center prospective study showed a promising response rate when combining etanercept with standard high-dose corticosteroids for first-line treatment of aGVHD compared with a cohort of contemporaneous case-matched patients treated with high-dose corticosteroids alone (61). However, the higher response rate observed with etanercept did not translate into a significantly superior survival at 6 months from aGVHD onset. Moreover, a multicenter prospective randomized “pick the winner” study comparing four promising molecules in combination with corticosteroids for first-line aGVHD treatment identified mycophenolate mofetil, and not etanercept, as the most promising agent (62). However, mycophenolate mofetil failed to prove any benefit in the subsequent multicenter, randomized, double-blinded, and placebo-controlled phase 3 trial evaluating its addition to standard corticosteroids (63). In the setting of steroid-refractory aGVHD, two small single center studies have shown only a modest effect of etanercept with few complete responses (64, 65). As for infliximab, efficacy seemed to be higher in case of gut involvement. Finally, a phase 2 study involving 100 patients also evaluated etanercept as part of aGVHD prophylaxis, in combination with tacrolimus and low-dose methotrexate (66). Once again, the benefit of etanercept was not obvious, as its addition to standard prophylaxis did not affect the overall risk of grade 2–4 aGVHD, as compared with a control cohort of 161 previously reported patients. Only a potential benefit among non-total-body-irradiated patients was suggested in this study. TNFR1 plasma level monitoring can also be used to evaluate and/or predict response to treatment with etanercept, as a significant reduction in these levels has been observed in responding patients (61, 66). To summarize, the current place of anti-TNF treatments in the arsenal of aGVHD is only limited to a therapeutic option for steroid-refractory disease, mostly in case of intestinal tract involvement. A possible explanation of this failure is that blocking TNF would also impact on the TNFR2-dependent Treg boost that is protective in GVHD as suggested by experimental data discussed below.

## Hope in Targeting TNFR2 (and Tregs) in GVHD

Regulatory T cells modulate alloreactivity during allo-SCT. Cell therapy using Tregs efficiently control GVHD (67, 68) whereas Treg deletion can be used to boost the graft-versus-leukemia (GVL) effect (69). Thus, some research teams envisioned TNFR2 as a potential target to act directly on Tregs in this setting and modulate alloreactivity with either TNFR2 agonists or antagonists. In this regard, three important studies were published almost simultaneously in 2016 (70–72).

In a murine model of aGVHD prevention relying on Treg infusion, we have clearly shown using three different experimental approaches that the protective effect mediated by therapeutic Tregs was dependent on TNF produced by pathogenic Tconv and TNFR2 expressed by Tregs (71). Indeed, when blocking the TNF/TNFR2 interaction with an anti-TNFR2 mAb, or when using either TNFR2-deficient therapeutic Tregs or TNF-deficient Tconvs, aGVHD prevention was abolished in all cases, highlighting a boost of alloreactivity after TNF/TNFR2 blockade. Moreover, Treg and Tconv phenotypes were also modified, with the former displaying decreased expression of activation and suppression markers while the latter showed increased production of pro-inflammatory cytokines.

The second study was published by Chopra and colleagues, who developed a TNFR2 agonist called STAR2 (70). *In vitro*, STAR2 was able to stimulate expansion and activation of Tregs, an effect not observed with Tconvs. This selective Treg expansion and activation was also triggered *in vivo*, when mice were treated with STAR2 intraperitoneal injections. Most of all, in a murine model of aGVHD, pretransplant administration of STAR2 to recipient mice protected from aGVHD and significantly increased survival. The protective effect of STAR2 was associated with a preserved GVL effect and had no deleterious effect on posttransplant anti-cytomegalovirus immune reconstitution.

Finally, in the study of Pierini and colleagues, therapeutic Tregs were preincubated *in vitro* with TNF (+IL-2) for a short period (72). This TNF priming resulted in a higher expression of Foxp3 and activation/suppression markers by Tregs and a higher proliferation rate. Most interestingly, when such “TNF-primed Tregs” were infused to recipient mice in an aGVHD murine model, this resulted in prolonged survival, increased weight gain, and improved GVHD clinical score, even at the very low 1:10 Treg:Tconv ratio. In this study also, the beneficial effect of TNF priming did not come with a detrimental loss of the GVL effect.

**Figure 3:**
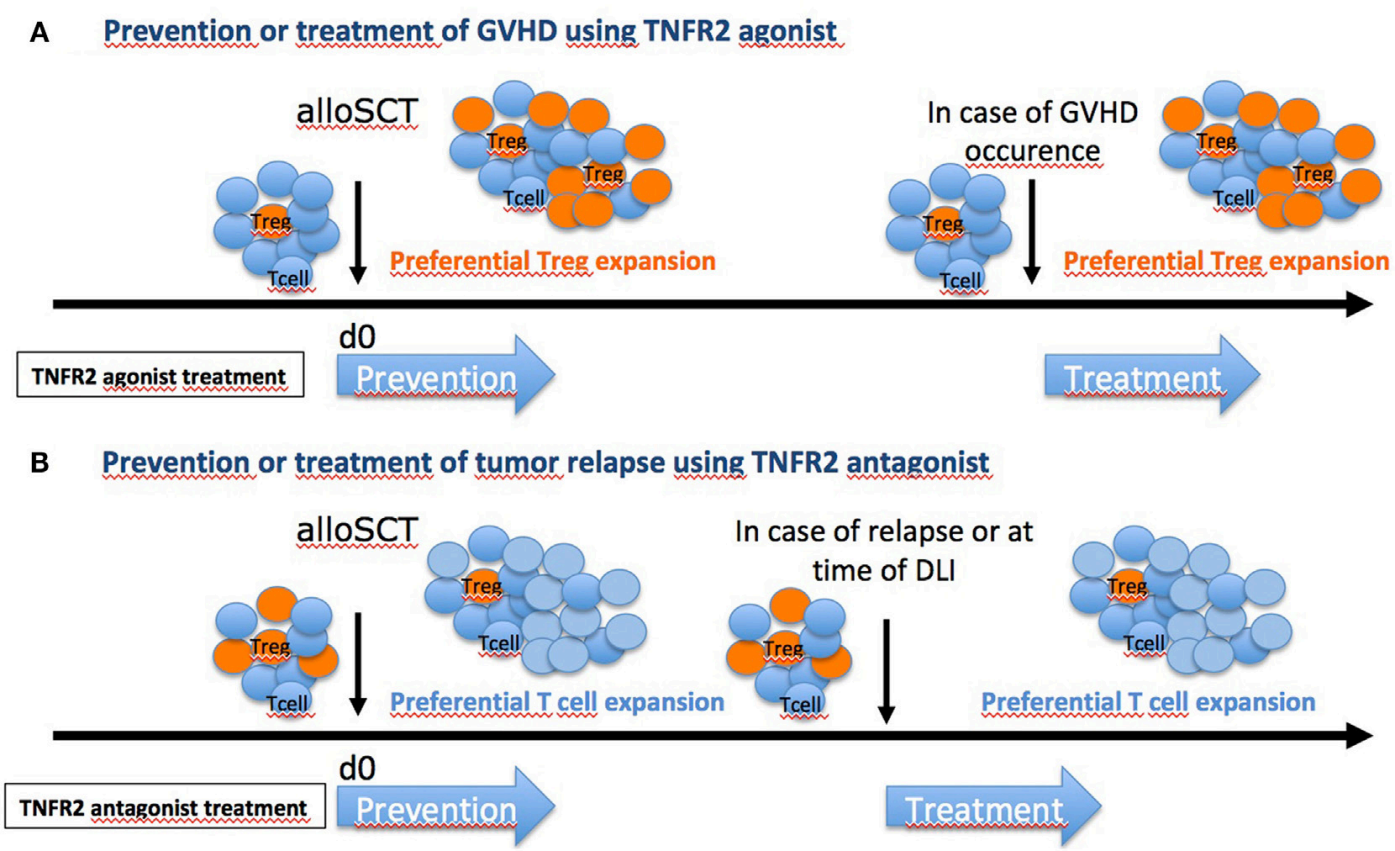
Hope in targeting TNFR2 [and regulatory T cells (Tregs)] in graft-versus-host disease (GVHD). Depending on the clinical situation and the risk for the patient to develop or not GVHD, different therapeutic strategies could be envisaged. **(A)** For patients with elevated risk of GVHD (unrelated donor or one or several HLA mismatch), TNFR2 agonist could be administered to recipients before allo-SCT, as shown previously (73), or at time of grafting to boost Tregs. Patients could also be treated at time of GVHD occurrence. **(B)** For patients with elevated risk of relapse (aggressive leukemia, geno-identical allo-SCT), anti-TNFR2 could be administered to recipients at time of grafting to inhibit Tregs. In case of tumor relapse, patients could also be treated at time of donor lymphocyte infusion (DLI) to block Treg effect.

Altogether, the results of these three studies pave the way for TNFR2 targeting to modulate alloreactivity after allo-SCT (Figure 3A). Additional preclinical data are needed, especially regarding the effect of TNFR2 agonists and antagonists on various human cell types (Tconvs, Tregs, and cancer cells) *in vitro*, before the start-up of clinical trials evaluating their efficacy and safety for prevention and/or treatment of aGVHD and posttransplant relapse of hematologic disease, respectively. Notably, in the setting of aGVHD prevention, single center clinical trials have shown the high potential of Treg cell therapy (73, 74). However, adoptive transfer of such cells is limited by the small proportion of Tregs among peripheral blood mononuclear cells (PBMCs) that necessitates an *ex vivo* culture for expansion before infusion to the patient. In this regard, the direct administration of a TNFR2 agonist to the patient to selectively activate and expand *in vivo* Tregs with the highest suppressive capacity holds the promise of a more simple, costless, less time consuming, and possibly more efficient method.

## New Checkpoint Inhibitors in Immunotherapy of Cancers and Role of Tregs

### New Targets in Immunotherapy of Cancers

**Figure 4:**
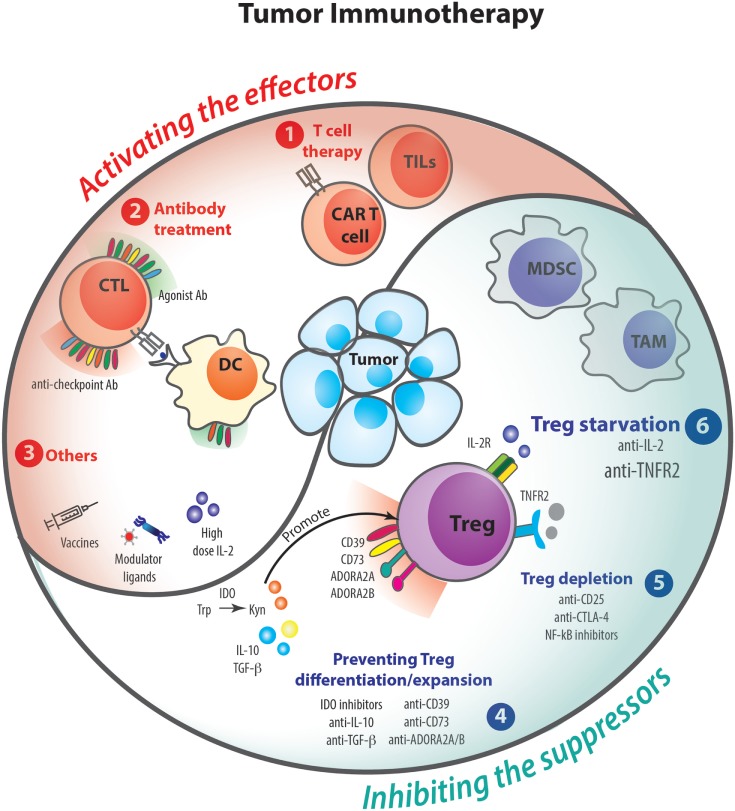
Tumor immunotherapies and regulatory T cells (Tregs). Over the past decades, several clinical trials and animal models have demonstrated that therapies acting on the immune response can help to fight against cancer. To control tumoral process, immunotherapies can either activate the effector arm of the immune response (1–3) or inhibit the suppressor mechanisms (4–6). The following therapies that potentiate T cell responses have been proposed: tumor-infiltrating lymphocytes (TILs) and CAR T cell thrapies (1); agonist and anti-checkpoint antibody treatments (2); other therapies such as vaccines, modulator ligands, and high doses of IL-2 (3). On the other hand, cancers promote suppressor mechanisms involving Tregs or myeloid-derived suppressor cell (MDSC), which are able to inhibit effector cells. Some treatments are being tested to modulate Treg suppression by preventing their differentiation/expansion (4), Treg depletion (5), or starvation (6).

Several clinical trials have clearly demonstrated that modulation of the immune response can improve the overall survival of advanced stage cancer patients (Figure 4) (75, 76). Indeed, since the approval of a-CTLA-4 antibody treatment for metastatic melanoma in 2011 (77–79), the field has witnessed the advent of numerous therapeutic approaches modulating the immune response (80, 81). Blockade of programmed death 1 and its major ligand PD-L1 has given impressive and durable clinical results (82–84) and fueled clinical evaluation (85) of (i) new inhibitory checkpoint targets, such as LAG-3 (86), TIM-3 (87), VISTA (88), and TIGIT (89), (ii) agonistic antibodies targeted to co-stimulatory receptors, such as 4-1BB (90), GITR (91), CD40 (92), and OX40 (93), (iii) cell-based therapies using dendritic cells, tumor-infiltrating lymphocytes (TILs), and genetically engineered T cells (CAR-T cells) (94), (iv) immune modulators such as innate ligands (95), and (v) vaccines, notably directed to neo-epitopes (94, 96). Along these lines, dozens of antitumor immunotherapeutic approaches have been already approved by regulatory agencies and thousands of such clinical trials are currently ongoing. Nevertheless, only 20–40% of patients benefit from these therapies, and some cases of resistance have been described (97–99).

Most of the abovementioned treatments are thought to work mainly by (re)-activating the cytotoxic arm of the immune response (100–102), namely, CD8+ T cells and NK cells; and by rescuing them from exhaustion (103, 104). Nevertheless, as the antitumoral immune response is also highly curtailed by Tregs, overcoming Treg-mediated immunosuppression in the tumor microenvironment (105, 106) represents a sound alternative for successful cancer immunotherapy. Of note, this can be obtained either by depleting Tregs or by inhibiting their function *in vivo* (107).

### Can We Treat Cancer by Depleting Tregs?

The first proof of the beneficial effect of Treg depletion on the antitumoral response was brought forward by Onizuka et al. (108). They showed that administration of an anti-CD25 antibody (mAb; PC61) had prophylactic, although not therapeutic efficacy, probably due to the concurrent elimination of CD25-expressing activated effector lymphocytes. More recently, the group of Quezada (109) has shown that anti-CD25 antibody-mediated Treg depletion can be ineffective due to the high expression of the inhibitory Fc receptor FcgRIIb by cells present in the tumor microenvironment. Consequently, anti-CD25 antibodies designed to avoid FcgRIIb binding induced massive Treg depletion in the tumor and led to impressive tumor regression. Also, specific depletion of Tregs in transgenic DEREG mice (110), which express a diphtheria toxin receptor under the control of the *Foxp3* regulatory sequences, resulted in a partial regression of established melanoma that correlated with CD8+ T cell accumulation in the tumor. Furthermore, mouse studies point out that anti-CTLA-4 antibodies mainly act by eliminating or inhibiting the tumor-associated Tregs (which highly express this molecule) rather than by reinvigorating exhausted T cells (111, 112). Indeed, controversial results have been observed with Daclizumab (an anti-CD25 antibody) and with a fusion protein between the IL-2 and the diphtheria toxin (Ontak) (113–118). Thus, direct proofs of the beneficial effect of Treg depletion in human are still missing for solid cancers, and there are to date no clinical tools that specifically target this population. This point is more advanced in the field of onco-hematology. With the intent of preventing or treating post allo-SCT relapse of hematologic disease, GVL effect can be activated by donor lymphocyte infusion (DLI). In this setting, the harmful effect of Tregs after DLI was suggested by a study in which the authors quantified Tregs in DLI products and demonstrated that patients with a durable complete remission of their malignancy after DLI had received a lower number of Tregs (119). This observation led to the idea of depleting Tregs to improve responses to DLIs, an approach that was successfully tested in a clinical trial in which a magnetic depletion of CD25+ cells was performed on donor PBMCs before their infusion to recipients that were considered “alloreactivity resistant” (69).

### Can We Treat Cancer by Modulating Treg Differentiation and Expansion?

Besides Treg depletion, tumor-associated Tregs can be therapeutically targeted by the modulation of the tumor microenvironment. Indeed, cancer cells produce metabolites, cytokines, and growth factors that can (i) promote Treg accumulation and expansion, (ii) enhance Treg function, and even (iii) induce Treg conversion from conventional CD4+ T cells (96). Among them, adenosine—generated upon catabolism of ATP by the ectoenzymes CD39 and CD73—and kynurenines—generated upon catabolism of tryptophan by the indoleamine 2,3-dioxygenase (IDO) enzyme—favor the accumulation, conversion, and expansion of Tregs and suppression of effector T cells (120). Accordingly, IDO inhibitors and either antagonists of A2A/A2B adenosine receptor or anti-CD39 and anti-CD73 antibodies significantly decrease the rate of Treg peripheral conversion and impair tumor growth (108, 121–124). Furthermore, therapeutic agents targeting these molecules in combination with immune checkpoint inhibitors show additive or synergistic effects in experimental tumor models, and their combination is currently under clinical investigation (96, 125). In addition, therapies aiming at inhibition of CD4+ T cell differentiation into Tregs have been tested. Among them, the effects of neutralizing antibodies or pharmacologic inhibitors of IL-10 and TGF-β have been evaluated in preclinical and clinical settings (126–128). These studies have demonstrated both pro- and antitumoral effects, probably due to their complex involvement in immune and non-immune processes. Moreover, there are not consistent data on the effect of these therapies on Tregs. Overall, manipulation of Treg induction and function through inhibition of metabolic and biochemical pathways active in the tumor microenvironment represent an alternative immunotherapeutic approach. Nevertheless, the significant side effects associated with the involvement of these pathways in different physiological processes must be taken into consideration.

### Can We Treat Cancer by IL-2 Deprivation to Target Tregs?

On top of the abovementioned strategies designed to disarm Tregs for therapeutic aims, “cytokine starvation or cytokine deviation” represents an alternative promising approach. Namely, deprivation of Tregs from IL-2 and TNF—two key cytokines essential for their biology—should lead to Treg dysfunction or death. Clinical manipulation of IL-2 levels remains complex as IL-2 can act both as an immune stimulating or suppressive cytokine, depending on the dose. On one hand, low-doses of IL-2 favor Treg survival and suppressive function and lead to a better control of autoimmune and inflammatory diseases (129–131). On the other hand, high-dose IL-2 administration boosts effector immunity and, consequently, enhances antiviral or antitumoral responses (132, 133). Noteworthy, in the cancer setting, low efficacy of high-dose IL-2 administration (134) can be explained in part by the unwanted effect of IL-2 on Tregs, which constitutively express the high affinity IL-2 receptor [composed by three subunits: IL-2-Rα (CD25), IL-2Rβ and IL-2Rγ] (135). For efficient antitumoral effect, there is a need to activate CD8+ T and NK cells, which also respond to IL-2 through the intermediate affinity IL-2 receptor, composed of IL-2Rβ and IL-2Rγ (136, 137). Interestingly, to prevent the IL-2 critical signal on Tregs, IL-2/anti-IL-2 antibody complexes, formed by an anti-IL-2 antibody acting as a CD25 mimotope hampering IL-2 fixation to CD25, were used to redirect IL-2 action to CD8+ T and NK cells (138). Of note, mutant IL-2 proteins have been designed to bear reduced binding affinity to CD25 and preserved affinity for IL-2Rβ, endowing them with preferential action on NK and CD8+ T cells. As for IL-2, depriving Tregs from TNF may also impair their function and improve antitumoral responses as detailed below. Thus, starvation of cytokine, such as IL-2, may emerge as a new firearm among the arsenal of immunotherapeutic strategies, which either alone or in combination, enrich the picture of immune checkpoint inhibitors available to fight cancers.

## Can We Treat Cancer by TNF Deprivation to Target Tregs?

### TNF Is Pro-Tumoral

As suggested by its name, TNF was described initially as a killer of cancer cells. We now know that this cytokine plays a complex role in cancer and tumor immunity because of its pleiotropic effect and the fact that it has two receptors. Actually, TNF is even considered mostly as a pro-tumor cytokine. Numerous mouse studies have shown that anti-TNF drugs reduced tumor growth in different types of cancers. This deleterious effect of TNF was further supported in TNF knockout mice that display reduced tumor growth (139–146). The individual role of TNFR1 and TNFR2 was assessed in knockout mice in some of these studies.

### TNF/TNFR1 Interaction Promotes Carcinogenesis and Pro-Tumoral Inflammation

**Figure 5:**
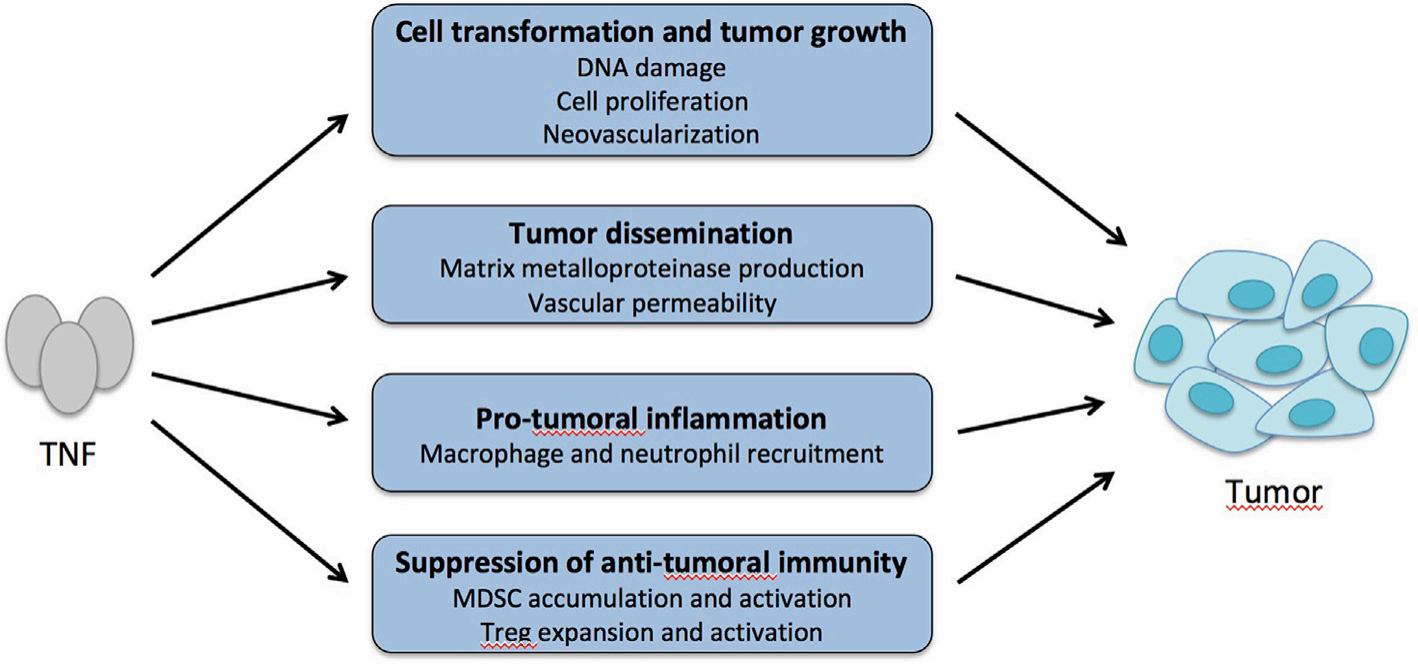
Tumor necrosis factor α (TNF) is a pro-tumoral cytokine. TNF may promote cell transformation and tumor growth by increasing DNA damage and mutations, abnormal cell proliferation, and neovascularization. TNF may also favor tumor cell dissemination by increasing matrix metalloproteinase production and vascular permeability and leakiness. By recruiting macrophages and neutrophils in the tumor environment that release inflammatory cytokines and chemokines, TNF may promote growth of tumors that respond to these inflammatory factors. Finally, by boosting the activity of myeloid-derived suppressor cells (MDSC) and regulatory T cells (Treg) in the tumor environment, TNF may indirectly suppress antitumor immunity.

The pro-tumoral effect of TNF has been explained by different mechanisms (Figure 5). TNF may directly promote cell transformation by activating oncogenes and inducing DNA damage (147). It may stimulate cell proliferation favoring cell transformation and neovascularization that is critical in cancer development (144, 146, 148). TNF may also promote growth of tumors that benefit from inflammatory cytokines and chemokines by recruiting neutrophils and macrophages (139, 141, 145, 149). Also, TNF may favor tumor invasiveness and metastasis by stimulating matrix metalloproteinase production and vascular permeability (150). When analyzed, the role of TNFR1 rather than TNFR2 was involved in these different mechanisms.

### TNF/TNFR2 Interaction Promotes Immunosuppression by Boosting MDSCs and Tregs

In mouse models of cancers, reduced tumor growth in mice treated by anti-TNF drugs or in TNFR2 knockout mice was associated with decreased numbers of MDSCs suggesting that TNF increases survival, recruitment, or function of MDSCs that suppress antitumor immunity (Figure 5) (20, 140, 143, 151). In a mouse model of melanoma, TNF injection favored tumor metastasis by acting on TNFR2-expressing hematopoietic cells, which was associated with an increase of Tregs (142). A similar mechanism was observed in models of colorectal cancer and hepatocarcinoma, since the tumor-dependent Treg expansion was abolished with an anti-TNFR2 mAb. Also, pretreatment of Tregs with TNF increased their capacity to suppress antitumor immunity after adoptive transfer (152).

In the setting of hematologic tumor relapse after allo-SCT, a similar approach using an anti-TNFR2 blocking mAb or TNFR2 antagonist could be considered to inactivate the deleterious effect of Tregs (Figure 3B). These molecules may be administered directly to the recipient to prevent or treat hematologic relapse, with or without a combined DLI, or even be used to preincubate donor PBMCs before infusion, to inactivate Tregs contained in the product.

### What about the Role of TNF in Cancer Patients?

All the above studies were performed in mice. What do we know about the role of TNF in cancer in patients? It is well described that some cancers, such as colorectal cancer and hepatocarcinoma, benefit from chronic inflammation. Importantly, recent meta-analyses of patients receiving anti-TNF treatment because of their autoimmune diseases did not show an increased risk of cancer development (153, 154). Also, because of the beneficial effect of anti-TNF administration in preclinical mouse models, some patients with advanced cancers received TNF blockers. In this phase II trial, infliximab and etanercept were well tolerated (155, 156). The possible effects of these treatments have been studied *in vitro* or *in vivo* after xeno-transplantation in immunodeficient mice. Results indicated that blocking TNF may reduce tumor growth, which is associated with reduced tumor dissemination, angiogenesis, and infiltration with myeloid cells (157–159). Finally, TNF may suppress antitumor immunity by boosting Tregs *via* TNFR2 since high amounts of TNFR2+ Tregs were associated with more severe lung and ovarian cancer (160, 161).

It has to be emphasized that these studies that provide possible mechanisms to explain the supratumoral effect of TNF were only based on correlations or *in vitro* observations. None of them has provided definitive *in vivo* proofs because of the pleiotropic effect of TNF. This would have required, for instance, conditional deletion of TNFR in a cell subset. However, based on what is known on the effect of TNF on Tregs and of Tregs on antitumoral immunity (see above), the possibility that TNF inhibits antitumor immunity by boosting Tregs is a very attractive hypothesis that may play a major role in some cancer types.

## Conclusive Remarks

Immunotherapy of cancers is a promising land but unfortunately only a minority of patients responds to these treatments. Among multiple targets that are being tested, TNFR2 is an attractive one. Indeed, TNF blockade may have different impacts by limiting cell transformation, neovascularization, or pro-tumoral inflammation and may boost antitumor immunity by acting on MDSC or Tregs. Recent works suggest that targeting TNFR2-expressing Tregs would be a safe and efficient way to stimulate antitumor immunity. Future experiments and clinical trials are required to validate this new therapy.

## Author Contributions

BS, EP, and JC organized the plan and structure of the manuscript, and all the authors contributed to the redaction.

## Conflict of Interest Statement

The authors declare that the research was conducted in the absence of any commercial or financial relationships that could be construed as a potential conflict of interest.
